# Machine Learning for Estimating Cardiorespiratory Fitness in Patients With Obesity: Protocol for a Retrospective and Prospective Multicenter Cohort Study

**DOI:** 10.2196/85069

**Published:** 2026-03-02

**Authors:** Jarle Berge, Vimala Nunavath, Rikke Aune Asbjørnsen, Heidi Borgeraas, Jens Kristoffer Hertel, Anita Dyb Linge, Inge Groote, Trine Stensrud, Espen Gjevestad, Linda Mathisen, Matthan WA Caan, Martin Paulson, Eva Maria Støa, Jan - Michael Johansen, AI and VO2max Group, Ida Husjord, Bjørn-Jostein Singstad

**Affiliations:** 1 Department of Endocrinology, Obesity and Nutrition, Vestfold Hospital Trust, Box 2168, Tonsberg, Norway, 47 33 34 41 11, 47 33 34 20 00; 2 Department of Neurology and Rehabilitation, Vestfold Hospital Trust, Stavern, Norway; 3 Department of Joint Operative Disciplines, Norwegian Police University College, Stavern, Norway; 4 Department of Science and Industry Systems, University of South-Eastern Norway, Kongsberg, Norway; 5 Department of Research and Innovation, Vestfold Hospital Trust, Tonsberg, Norway; 6 Departement of Digital Health Research, Oslo University Hospital, Oslo, Norway; 7 Muritunet Rehabilitation Centre, Valldal, Norway; 8 Department of Social Science and History, Volda University College, Volda, Norway; 9 Department of Radiology, Vestfold Hospital Trust, Tonsberg, Norway; 10 Department of Sports Medicine, Norwegian School of Sports Sciences, Oslo, Norway; 11 Department of Radiology, University of Amsterdam, Amsterdam, The Netherlands; 12 Department of Oncology and Hematology, Vestfold Hospital Trust, Tonsberg, Norway; 13 Department of Sports, Physical Education and Outdoor Studies, University of South-Eastern Norway, Bø, Norway; 14 See Acknowledgments; 15 Medical Technology and E-health, Akershus University Hospital, Lørenskog, Norway; 16 Institute of Clinical Medicine, University of Oslo, Oslo, Norway

**Keywords:** VO2max, VO2max estimation, regression, obesity, cardiorespiratory fitness, cardiorespiratory fitness estimation, machine learning, web application

## Abstract

**Background:**

Cardiorespiratory fitness (CRF) is a key predictor of cardiovascular and other health-related diseases in individuals with obesity. CRF is most accurately assessed through maximal exercise testing with advanced gas-analysis equipment (maximum volume of oxygen [VO_2max_]); however, this approach is time-consuming, costly, and requires specialized expertise. Therefore, submaximal tests and self-reported physical activity levels have been used to develop predictive algorithms to estimate CRF, yet they often performed poorly in individuals with low CRF levels, such as patients with obesity, because they are predominantly developed using data from healthy populations. Studies using machine learning (ML) models based on VO_2max_ data from patients with obesity appear to be lacking in the literature. ML models based on routinely collected clinical measures may offer a more practical and potentially accurate way to estimate CRF, reducing time, costs, and clinical burden.

**Objective:**

The primary aim of this study is to use multicenter, longitudinal, real-world clinical data from a uniquely characterized population with obesity to develop and validate a clinically relevant ML model for estimating CRF and to compare its performance with the gold standard of VO_2max_ testing.

**Methods:**

A retrospective data set combining assessments of VO_2max_ tests and clinical parameters from adult patients with severe obesity BMI (≥40.0 kg/m^2^ or 35.0‐39.9 kg/m^2^ with at least 1 obesity-related comorbidity) from Vestfold Hospital Trust, Muritunet Rehabilitation Institution, and Norwegian School of Sport Sciences will be the foundation for developing the ML model. The clinically relevant ML model for estimating CRF will be presented as a web application, allowing easy access and interaction. The model’s estimations will be compared against direct VO_2max_ measurements obtained from medical equipment across institutions as part of a prospective validation. Ethical approval has been obtained for the use of 2 databases in the initial model development; approval for the remaining data and prospective phase is pending.

**Results:**

Vestfold Hospital Trust, the Norwegian School of Sport Medicine, and Muritunet Rehabilitation Institution conducted more than 2623 VO_₂max_ tests and collected clinical parameters from 1279 adults with severe obesity during 2013-2025, both before, during, and after lifestyle interventions. The first scientific publication of the clinically relevant ML model is expected to be published in 2026. The results of the overall project are expected to be completed in 2028. The project was awarded salary funding in two rounds from Vestfold Hospital (October and December 2023) and salary funding from the Research Council of Norway (October 2024). In addition, the project received allocated supervision hours from InnoMed Norway (April 2024).

**Conclusions:**

This project aims to develop a clinically relevant ML model, which serves as a cost-effective tool for CRF estimation in individuals with obesity, improving accessibility to this important health marker. To our knowledge, this is the first initiative in Norway to estimate CRF in individuals with obesity using ML, based on a unique clinical database. The project carries substantial societal value and holds national and international relevance for health care practice and patient outcomes.

## Introduction

### Background

Obesity is a primary risk factor for more than 2.8 million preventable deaths worldwide [1] and over 3200 preventable deaths in Norway [2]. Obesity is also strongly linked to lost years of life [2], primarily due to cardiovascular disease and other obesity-related diseases [3-7]. Cardiovascular disease is closely related to cardiorespiratory fitness (CRF), defined as the body’s ability to use oxygen [8]. Further, obesity is associated with poor physical ability and impaired CRF. Therefore, improvements in cardiorespiratory fitness reduce the risk of cardiovascular disease, all-cause mortality, and contribute to enhanced overall health among people with obesity [7,9]. CRF is thus an important health indicator for both the prevention and treatment of obesity [6,10,11].

The gold standard for measurement of CRF is a maximal test on a treadmill or bicycle connected to expensive medical equipment that analyzes respiratory gas (maximum volume of oxygen [VO_2max_, [mL*min^−1^]) [12,13]. Performing the test is a burden for the patients related to travel, time, absence from work or school, and costs (eg, parking and travel cost). The challenges for the clinic are financial costs, time investment, and the burden of administrating a direct physical measurement of the VO_2max_ test. In addition, the test requires experience and knowledge of a test leader [13]. Further, methods for indirect estimation of VO_2max_ or more precise assessment of CRF have been developed, such as the “submaximal tests” [14,15]. The submaximal assessment of CRF does not require patients to exercise to exhaustion but instead estimates CRF based on physiological responses, usually heart rate, speed, intensity, and duration during exercise on a treadmill, ergometer cycle, or step box [13,16]. A further simplification is self-reported questionnaires that include information on physical activity level, demographic characteristics, and clinical characteristics have also been used for estimating CRF alone or in combination with submaximal assessments [13,17]. Nevertheless, the challenges associated with both the direct maximal test and submaximal test have contributed to the development of different predictive algorithms to estimate CRF [16-18]. The algorithms are developed using data from direct physical measurements of VO_2max_ and commonly require information about gender, age, height, body weight, heart rate (maximal or resting values), and exercise habits to estimate CRF [16,17]. These algorithms are typically developed using conventional statistical models and are generally advantageous and convenient for large populations [16,17]. Furthermore, as algorithms are commonly trained on data from healthy volunteers, the lack of sufficient data from individuals with low VO_2max_ values (eg, patients with obesity) may lead to less reliable estimates [13,16,17,19,20].

In recent years, machine learning (ML) has demonstrated that advanced models can outperform conventional statistical models in clinical prediction tasks. A ML model is a mathematical construct trained to recognize patterns in data, enabling it to make predictions or informed decisions based on nonlinear combinations of input variables, without requiring explicit programming for each specific task. Therefore, ML may provide more accurate estimates of CRF compared to traditional methods. A digital accessibility solution incorporating a ML model with high accuracy for estimating CRF could enable routine assessment of this key health indicator in clinical settings for patients with obesity [18]. This, in turn, may facilitate the integration of CRF into broader clinical decision-making and evaluation of individuals’ health.

### Objectives

Studies using ML models based on VO_2max_ data from patients with overweight and obesity appear to be lacking in the literature [13]. Therefore, the primary objective of this study is to use multicenter, longitudinal, real-world clinical data from a uniquely characterized population with obesity to develop and validate a clinically relevant ML model for estimating CRF, and to compare its performance with the gold standard of VO_2max_ testing. Furthermore, the model will be implemented as a web-based tool to support real-world use in a European clinical setting. Initial feedback and empirical insights into the usability and feasibility of the web application will be collected in health care contexts. The primary hypothesis is that a clinically relevant ML model can estimate CRF with acceptable accuracy. To accomplish this, the following subgoals have been established:

1. Model development and evaluation of retrospective data: Train and evaluate multiple advanced ML models using our retrospective dataset of VO_2max_ and other medical measurements (eg, body weight, age, and fat mass) to estimate CRF in individuals with obesity and select the top-performing model.
2. Web application development: Develop a web-based application that incorporates the optimal ML model identified in goal 1, to enable accessible VO_2max_ estimation.
3. Prospective validation and pilot study: Conduct a prospective validation study using the web application, developed in goal 2, to evaluate the performance of the ML model. This will involve comparing the model’s predictions of CRF with direct physical measurements of VO_2max_. Additionally, a pilot study will be carried out to gather initial feedback and insights on the usability and feasibility of the web application within the health care setting.
4. Model management and monitoring: Implement a system to manage and monitor the ML model, aligning with relevant regulatory frameworks like the European Union Artificial Intelligence Act [21]. This system will include mechanisms for detecting and addressing out-of-distribution data, as well as provisions for continuous model re-training using data routinely collected in the clinical setting.
5. Implementation in clinical practice*:* Implement the ML model estimation through the web application as a clinical tool.

## Methods

### Machine Learning Model Development and Prospective Validation

ML models differ from simpler statistical approaches by their ability to capture nonlinear relationships within data, unlike traditional statistical methods that focus on linear associations. The capacity of ML models to uncover nonlinear patterns enables them to approximate a wide range of mathematical functions, revealing relationships that would be difficult to discern using classical statistical techniques. Therefore, in the retrospective development phase, the model will be trained and internally evaluated using existing, previously collected measurements, both published and unpublished data. In the prospective validation phase, the final model will be evaluated in a prospective validation to compare its predictions to real-world, directly physically measured VO_2max_ values in a different health care setting. Concurrently with the prospective validation, a pilot study will be conducted to assess the feasibility, usability, and integration of the web application into clinical workflows.

### Study Design

This protocol describes both a retrospective multicenter predictive modeling study and a prospective multicenter observational cohort study.

The study will be developed in accordance with the [22,22] extension, Transparent Reporting of a multivariable prediction model for Individual Prognosis Or Diagnosis plus Artificial Intelligence extension 2024 statement [23] and the CONSORT (Consolidated Standards of Reporting Trials)-EHEALTH [24]. The CONSORT-AI guideline ensures transparent reporting of artificial intelligence (AI)-based interventions in the trial, including algorithm version, input data handling, and human–AI interaction. Transparent Reporting of a multivariable prediction model for Individual Prognosis Or Diagnosis-AI is used to guide reporting of the ML prediction model’s development, validation, and performance. CONSORT-EHEALTH is applied to capture key digital intervention details such as delivery platform, user interaction, and data flow. Further, the study follows the SPIRIT (Standard Protocol Items: Recommendations for Interventional Trials)-AI checklist (Checklist 1) for clinical trial protocols involving AI [25]. Together, these guidelines support clarity, reproducibility, and critical appraisal of our AI-driven digital health evaluation.

### Participants and Data Collection

#### Inclusion Criteria

The inclusion criteria for both the retrospective and prospective components of this study are patients (≥18 y) diagnosed with severe obesity, which corresponds to BMI greater than or equal to 40.0 kg/m^2^, or 35.0‐39.9 kg/m^2^ with at least 1 obesity-related comorbidity (eg, type 2 diabetes, hypertension, dyslipidemia, or obstructive sleep apnea). Additionally, participants who completed 1 VO_2max_ test at either baseline, 9, 12, 17, 25 weeks, or at 1 to 2 years were included in the retrospective cohort study.

#### Exclusion Criteria

Patients will be excluded from the study if they are aged younger than 18 years or have a BMI below 35.0 kg/m². Patients who have not completed at least 1 VO_2max_ test at any of the specified assessment points (baseline, wk 9, 12, 17, 25, or at 1 to 2 y) will be ineligible. Further, patients with incomplete or insufficient medical measurements of VO_2max_ data will likewise be excluded.

### Retrospective Study: Subgoal 1

In the retrospective study, an accurate estimation of CRF using ML models will be developed, specifically tailored to individuals with obesity. ML models will be trained on datasets combining results from direct physical assessments of VO_2max_ and relevant clinical parameters derived from patients with obesity. The retrospective study will use data collected at Vestfold Hospital Trust (VHT), Norwegian School of Sport Sciences (NIH), and Muritunet Rehabilitation Institution (MRI) to train a ML model.

VHT possesses a dataset comprising more than 700 adults with severe obesity. The data was collected in 3 different research studies approved by the Regional Committees for Medical and Health Research Ethics South East, Norway.

The first study was a randomized controlled trial (REK ID: 2013/1849, Clinical Trials NCT02311738) [26]. Data from 73 patients seeking treatment at the Department of Endocrinology, Obesity, and Nutrition was collected between January 5, 2015, and June 9, 2017. A total of 243 VO_2max_ tests were performed at baseline and at approximately 9 weeks, 17 weeks, and 25 weeks (Table 1).

The second study was a retrospective cohort study (REK: 2016/1414, ClinicalTrials.gov: NCT03593798) [19]. Data from 180 patients seeking treatment at the Clinic of Physical Medicine and Rehabilitation were collected between November 1, 2013, and January 1, 2017. A total of 442 VO_2max_ tests were performed at baseline and at approximately 12-week and 1-year follow-ups (Table 1).

The third study is an ongoing retrospective cohort study (REK: 2017/88, Clinical Trials: NCT03593148) started in May 2017 and will end in June 2026. Data thus far have been collected from 453 patients seeking treatment at the Clinic of Physical Medicine and Rehabilitation. A total of1400 or more VO_2max_ tests were performed at baseline and approximately 12-week, 14-week, 24-week, 1-year, and 2-year follow-ups (Table 1).

**Table 1.** Test time of maximum volume of oxygen.

Test time	Baseline	9 wk	12 wk	17 wk	25 wk	1 y	2 y
Study 1 at VHT^a^	✓^b^	✓		✓	✓		
Study 2 at VHT	✓		✓			✓	
Study 3 at VHT	✓		✓			✓	✓
Study 4 at NIH^c^	✓		✓		✓		
Clinical registry 5 at MRI^d^	✓				✓	✓	

a VHT: Vestfold Hospital Trust.

b ✓: data available at this measuring point.

c NIH: Norwegian School of Sport Sciences.

d MRI: Muritunet Rehabilitation Institution.

NIH has collected data from 43 patients with obesity through 2 projects.

NIH has collected data from patients with obesity in 2012 and 2013. The projects were part of a larger PhD project aiming to evaluate longitudinal lifestyle changes in obese patients (REK Sør-Øst C-2009/1699). A total of 101 VO_2max_ tests were performed at baseline to 12 and 25 weeks after the intervention (Table 1).

MRI in Møre and Romsdal, Norway, has collected data from more than 750 patients with obesity.

Muritunet has a clinical quality registry with consent for research (Norwegian Data Protection Authority 17/00433‐2/SBO). The clinical quality registry started on May 16, 2017, and is still ongoing. More than 1300 VO_2max_ tests were performed at baseline to approximately 6- and 12-month follow-up (Table 1).

VHT, NIH, and MRI together provide a comprehensive dataset from more than 1500 adult patients with severe obesity and more than 3600 completed VO_2max_ tests. This diverse data source allows for the development of a robust ML model capable of accurately estimating VO_2max_ in patients with obesity.

### Medical Measurements

#### Brief Summary of All Medical Measurements

The medical measures obtained from studies were maximal oxygen consumption, body weight, age, sex, BMI, height, waist and hip circumferences, fat-free mass and fat mass, diastolic and systolic blood pressure, heart rate maximum, respiratory quotient, resting oxygen saturation, oxygen saturation before test, speed or watt, incline and total time duration on treadmill, resting heart rate, and Borg Scale. Self-reported questionnaire of generic measure of health-related quality of life (Short Form Health Survey, SF-36) [27,28], obesity-specific measure of health-related quality of life [29], and a symptom measure of obesity (weight-related symptom measure of obesity) will also be used [30,31] (Table 2).

**Table 2.** Parameters collected from different studies or registries.

Parameters	Study 1 (VHT^a^)	Study 2 (VHT)	Study 3 (VHT)	Study 4 (NIH^b^)	Clinical registry 5 (MRI^c^)
Sex (male/female)	✓^d^	✓	✓	✓	✓
Age (y)	✓	✓	✓	✓	✓
Height (cm)	✓	✓	✓	✓	✓
Body weight (kg)	✓	✓	✓	✓	✓
Maximal volume of oxygen (VO_2max_, mL•min^-1^)	✓	✓	✓	✓	✓
BMI (kg/m^2^)	✓	✓	✓	✓	✓
Waist circumferences (cm)	✓	✓	✓	✓	✓
Hip circumferences (cm)	✓	✓	✓	✓	✓
Fat mass (kg)	✓	✓	✓	✓	✓
Fat-free mass (kg)	✓	✓	✓	✓	✓
Systolic blood pressure (mm Hg)	✓	✓	✓	✓	
Diastolic blood pressure (mm Hg)	✓	✓	✓	✓	
Resting heart rate (bpm)	✓	✓	✓	✓	
Heart rate maximum (bpm)	✓	✓	✓	✓	
Respiratory exchange ratio	✓	✓	✓	✓	
Oxygen saturation before test (%)				✓	
Ventilation maximum (mL•min^-1^)				✓	
Speed treadmill (km/t)	✓	✓	✓		
Watt bike (W)					
Incline treadmill (%)	✓	✓	✓		
Speed or watt ergometer bike (w)		✓	✓		
Total time duration treadmill (min)	✓	✓	✓		
Total time bike (min)		✓			
SF-36^e^	✓		✓		
IWQOL-Lite^f^	✓		✓		
WRSM^g^	✓		✓		
Sociodemographic data	✓	✓	✓	✓	✓
Other relevant health parameters	✓	✓	✓	✓	✓

a VHT: Vestfold Hospital Trust.

b NIH: Norwegian School of Sport Sciences.

c MRI: Muritunet Rehabilitation Institution.

d ✓: data available at this measuring point.

e SF-36: health-related quality of life was measured with the Short Form Health Survey.

f IWQOL-Lite: impact of weight on quality of life.

g WRSM: weight-related symptom measure of obesity.

#### Maximal Volume of Oxygen

The VO_2max_ was expressed in absolute values as liters per min (mL/min) and often presented as relative to body weight (mL/kg/min) as CRF [8]. The VO_2max_ was measured by the use of Metalyser, Cortex 2 (Biophysik) (study 1), Jaeger Oxycon Pro ergospirometry test system (JLAB 5.x) (study 2 and 3), and Vyntus CPX metabolic system (Vyaire Medical) (part of study 3 [gen. 3.20] and 4 [gen. 1]). Metalyser cortex 2 has an accuracy of 4%‐5%, Jaeger Oxycon Pro of 3%, and Vyntus CPX 3% compared to mechanical lung. Before each ergo test, the device was calibrated with room air (23 °C) at 20.93% O_2_ and 0.03% CO_2_ and a certified gas containing 15.80% O_2_ and 4.98% CO_2_. The calibration of ventilatory volume was conducted with a manual 3-liter syringe (Hans Rudolph, Shawnee, KS, USA). Barometric pressure was calibrated with an electronic barometer (GB3300 Greisinger electronic) or automatically by the system. A face mask (Hans Rudolph V2) with different sizes (petit, XS, S, M, L) was used to collect expired air during the tests. The Metalyser, Cortex 2, and Jaeger Oxycon Pro ergospirometry system was equipped with a mixing chamber, with O_2_, and CO_2_, and the ventilation was analyzed continuously. Vyntus CPX metabolic was equipped with breath-by-breath open system.

#### Maximal Volume of Oxygen Test

The test was performed as an individualized incremental treadmill test or modified Balke protocol on a Woodway PPS 55 plus or ELG 5, for patients with walking restrictions (n=8), as an incremental bicycle test (Lode Corival V3, Lode BV).

For the individualized incremental treadmill test at VHT, velocity was increased alternately by 0.5 km/h, or the inclination by 1% every 30 seconds until voluntary exhaustion. The modified Balke protocol at NIH starts at 3 minutes at 4.5 km/h and 4 % inclination, the next level was 4.8 km/h and 6% inclination, and thereafter 2% inclination every minute up to 20%. The modified Balke protocol at MRI starts at 2 minutes at 3.6 km/h and increases 0.6 km/h every 2 minutes until 6 minutes. Then the speed was standing at 5.4 km/h with an inclination of 4%, and thereafter 2% inclination every minute up to 20%. If the patient could bear to continue after the maximal incline, the speed was increased by 0.5/h every minute until exhaustion.

For the bicycle test, brake power was increased by 25 watts every 30 seconds until voluntary exhaustion. The duration of the tests ranged from 4 to 10 minutes.

Two or more of the following criteria must be met for a valid VO_2max_ test; voluntary exhaustion, respiratory exchange ratio greater than or equal to 1.05, heart rate greater than or equal to 95% of heart rate maximum, Borg scale greater than or equal to 17, and a flattening of the VO_2_ curve (mL/min) were used to evaluate if VO_2max_ had been achieved. VO_2max_ was calculated as the highest of 3 consecutive 10 seconds measurements (total 30 s) or the highest of 6 consecutive 5 seconds measurements (total 30 s).

#### Heart Rate Maximum

Heart rate maximum was measured automatically every 5 seconds with Polar WearLink + H7 bluetooth or manually each minute (Polar Electro OY) and set as the highest observed value during the test.

#### Speed and Incline on Treadmill

Speed (km/h) and incline (%) were set as the maximum reported value during the test on treadmill.

#### Respiratory Exchange Ratio

Respiratory exchange ratio measures the ratio of carbon dioxide produced (VCO₂) to oxygen consumed (VO₂) during VO_2max_ test. The respiratory exchange ratio was measured with the same equipment as used to measure VO_2max_.

#### Body Weight

Body weight (kg) was measured with patients wearing light clothing and no shoes on Scanvaegt DS-530 (study 2 and 3), or with the bioelectrical impedance analyzer Tanita (MC-780 or BC-418), or Inbody 720, (100‐240 W).

#### Height

Height (cm) was measured to the nearest 0.1 cm using Soehnle professional stadiometer.

#### BMI Calculation

BMI was calculated as weight in kilograms divided by height in meters squared (kg/m^2^).

#### Waist Circumference

Waist circumference (cm) was measured to the nearest 0.1 cm using a measuring tape placed horizontally at the midpoint between the lower margin of the lowest rib and the top of the iliac crest, with the abdomen relaxed.

#### Hip Circumference

Hip circumference (cm) was measured using a measuring tape placed around the widest part of the hips, with participants standing upright and with their feet together.

#### Fat Mass and Fat-Free Mass

Body composition as fat mass (kg) and fat-free mass (kg) was measured with patients wearing light clothing and no shoes or socks using the bioelectrical impedance analyzer Tanita BC-418 or Inbody 720, (100‐240 W).

#### Blood Pressure

Blood pressure (mm Hg) was measured using an automatic sphygmomanometer (Dinamap V100). The person was seated in a relaxed position with the arm resting on the table. Three measurements were collected, and the average of these 3 was used.

#### Resting Heart Rate

The resting heart rate was measured at the same time as the blood pressure, and the average of 3 measurements was used.

### Self-Reported Measurements

#### Health-Related Quality of Life

The generic measure of health-related quality of life was measured with the Short Form Health Survey (SF-36, version 2.0), a 36-item instrument designed to assess generic health-related quality of life. Of these, 35 items are used to generate scores across 8 dimensions: physical functioning, role limitations due to physical problems, bodily pain, general health, vitality, social functioning, role limitations due to emotional problems, and mental health [27,28]. Each dimension is scored on a scale from 0 to 100, with higher scores indicating better health-related quality of life. The SF-36 has been translated into Norwegian [32] and has shown strong psychometric properties across various medical conditions [32-35], including among patients with severe obesity [36].

#### Obesity-Specific Measure of Health-Related Quality of Life

The obesity-specific measure of impact of weight on quality of life was measured with a 31-item questionnaire designed to assess weight-related quality of life [29]. Four domain scores were evaluated: physical function, self-esteem, sexual life, and public distress. Each domain is scored on a scale from 0 to 100, with lower scores reflecting greater impairment.

#### Symptom Measures of Obesity

The weight-related symptom measure of obesity was measured with a 20-item for the presence and distress of 20 weight-related symptoms [30,31]. The symptoms are scored on a 6-point Likert scale. The scale ranges from 0 (does not bother at all) to 6 (bothers a very great deal) with higher scores indicating worse symptom distress.

### Web Application: Subgoal 2

The web application design will be developed by Natalia Kaur together with the “AI and VO_2max_” group. An application programming interface that serves as the interface facilitating communication between a data system and an external service or system will be used in the web application. It enables the exchange of queries, whereby the external system processes incoming requests and provides a corresponding response. Figma will be used to develop a user-centric interface prototype in an agile manner. The finalized design will then be implemented as a .NET application and hosted on Microsoft Azure, a cloud-based platform that supports seamless deployment and scalable web applications. Our Python-based ML model will be hosted on a web server and made accessible to the web application through an application programming interface, enabling seamless communication with Microsoft Azure and our front-end application. Practitioners and patients will actively contribute to the design, development, testing, and feedback of the web application through an agile, interdisciplinary development team assembled for the project. This collaborative approach will ensure the implementation of a user-friendly web application (subgoal 2), continuing the focus on the user interface process.

### Prospective Study: Subgoal 3

In the prospective validation, the results of the ML-based CRF estimation model in the web application will be compared with direct physical measurements of VO_2max_ obtained using cardiopulmonary exercise testing equipment for testing from different institutions. In each project participating institution, a principal investigator with expertise in physical measurements of direct VO_2max_ will be responsible for the coordination of the prospective study, together with the project leader (JB).

Patients who meet the eligibility criteria of overweight and obese will be given both verbal and written information of the prospective study. The participant will be given up to 1 week to consider their participation. If they agree to be included in the prospective study, written informed consent will be obtained from all patients.

Once enrolled, participants will undergo direct physical measurements of VO_2max_ with medical equipment, and simultaneously, the principal investigator will collect all necessary input parameters required for the ML-based model. The ML model will generate an estimated CRF with the web application, which will be recorded alongside the measured VO_2max_ result. All data, including participant details, model inputs, model output, and corresponding direct physical measurements of VO_2max_, will be entered into a secure, study-specific database.

#### Testing and Iterative Improvement

The web application will be tested for user experience in both primary and secondary health care settings, focusing on usefulness, usability, and engagement to support iterative improvements. A study-specific questionnaire will be used (eg, technology acceptance model or system usability scale). The diverse project partners will actively contribute to its implementation across these health care sectors.

### Model Management and Monitoring: Subgoal 4

To develop a robust ML model, we will continuously retrain it using new VO_2max_, measurements, along with anthropometric and demographic data from the health care clinics. Each retrained model will be saved (version control) and tested immediately after retraining to assess the performance and control for unintentional performance drop. The history of model retraining and performance will be maintained and made accessible to system administrators via a dashboard.

### Implementation: Subgoal 5

The ML model estimation through the web application will be implemented as a clinical tool, and thereby CRF may be used as a diagnostic and prognostic health indicator. The clinical tool will support health care professionals in hospitals, rehabilitation centers, primary care services, general practice, and in other settings. This clinical tool will enable faster, more targeted, and personalized treatment while reducing the workload on health care services by eliminating the need for direct physical measurement of VO_2max_.

### Statistical Analysis

#### User Statistics

The statistical analysis will be performed using Python (3.12.12; Python Software Foundation). Categorical variables will be summarized as frequencies and percentages, and their comparisons will be conducted using the chi-square test. For continuous variables, analyses will be conducted using independent samples *t* tests or Mann-Whitney *U* tests, with results presented as mean (SD) for normally distributed data or median (IQR) for nonnormally distributed data. To estimate the variance and 95% CI of the model’s performance on the test set, bootstrap resampling will be performed.

#### Sample Size Calculation

In traditional linear statistics, a common rule of thumb is that at least 10‐20 observations per predictor are required to ensure stable model estimation [37]. However, in our study, we plan to apply more advanced ML models (such as random forest regression), which are likely to require substantially larger sample sizes to avoid overfitting and to achieve reliable generalization. In addition, we expect a considerable amount of missing data across the participating centers, which further increases the need for a larger dataset. For these reasons, we plan to include all available data from the study centers. This approach allows for a fair comparison between simpler regression models and more complex ML models, while also ensuring sufficient statistical power despite missing data and model complexity.

For the prospective study, we performed a sample size calculation using 1-sample mean test, shown in Equation 1, where $Z_{\beta }$ represents the standard normal deviate corresponding to the desired statistical power. We selected 80% power, which is commonly used in clinical research, resulting in $Z_{\beta }$=0.84. The SD of VO_2max_ was used. In our population, estimated using data from 2 of the databases included in this study, this value was found to be 23% of the mean.

**Figure 1.**
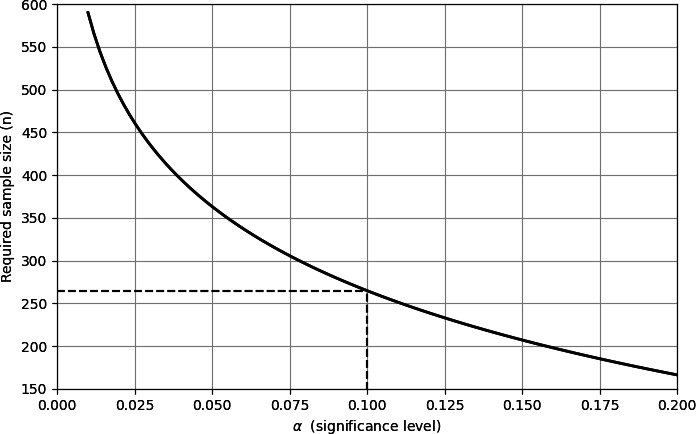
Sample size (n) for different significance levels α.

The parameter σ represents the acceptable clinical error and was set to 3%, based on the established measurement error of the gold standard method. $Z_{\alpha }$ corresponds to the selected significance level. A 1-sided test was selected under the assumption that the model would outperform random prediction given the population distribution. Figure 1 shows the required sample size for different significance levels (α), while keeping the other parameters fixed as defined above.

The selected significance level for our study is *α*=.10, which corresponds to a 1-sided 90% confidence level. Under these assumptions, the required sample size is 265 patients, enabling us to detect an improvement that exceeds the acceptable error threshold with 80% power.


(Eq.1) $$n={\left(\frac{Z_{\alpha }+Z_{\beta }}{\frac{\sigma }{SD}}\right)}^{2}$$

#### Model Architecture

Candidate algorithms were chosen to represent a broad range of linear, kernel-based, and tree-based regression approaches. These approaches included linear models (ordinary least squares linear regression, ridge regression, least absolute shrinkage and selection operator, and elastic net regression), Bayesian models (Bayesian ridge regression and automatic relevance determination regression), support vector regression models (linear support vector regression and nu-support vector regression), and tree-based ensemble models (decision tree regressor, extremely randomized trees regression, random forest regression, and histogram-based gradient boosting regression).

#### Data Preprocessing

A sequential data preprocessing pipeline will be implemented, consisting of three primary components: (1) missing value imputation, (2) feature standardization, and (3) input to regression model. All preprocessing steps will be incorporated into a unified pipeline to ensure robustness and reproducibility.

To address incomplete data in model development and testing, missing values will be imputed using the k-nearest neighbor imputation method. The k-nearest neighbor algorithm will identify the set of k-nearest neighbors based on Euclidean distance metrics computed over the available (observed) feature values and impute missing values using the mean of these neighbors.

To ensure that features contribute proportionally to model training and to improve model convergence, feature standardization will be applied. Continuous predictors will be rescaled using *z*-score normalization. The estimated scaling parameters, trained on the training folds or the development set, will then be applied to the corresponding validation and test sets.

#### Model Development and Evaluation

Model development will be performed with Python (3.12.12) and packages such as scikit-learn (version 1.6.0; scikit-learn developers). Demographic and anthropometric parameters (Table 2) from the datasets obtained from VHT, MRI, and the NIH will be divided into a development set (80%) and a test set (20%), using a patient-level grouped split strategy to ensure that all measurements from a given patient are included in only 1 of the 2 sets. This approach will be used to prevent data leakage between the development and test sets.

The development set will be used to perform a structured hyperparameter search for the optimal configuration of the model. The final model will be selected based on cross-validation, and the best-performing model across the k-folds on the development set will be used for final testing on the test set. As with the development–test split, the *k*-fold cross-validation will be grouped at the patient level to ensure that all measurements from the same patient are included in only 1 fold, thereby preventing data leakage.

During the development phase, we will also experiment with different stratification strategies related to the center of origin of the dataset, to identify potential bias, including systematic differences arising from variations in measurement protocols between centers.

The performance on the test set will be reported in terms of metrics such as *R*-squared score, root mean squared error, mean absolute error and mean absolute percentage error, standard error of estimate (SEE), and SEE given in percentage. In addition to the CRF estimation model, a pipeline of preprocessing steps will be constructed, doing necessary things such as feature scaling and imputation. The ML model with the best performance (subgoal 1) will be implemented in the web application (subgoal 2) and further used in the prospective study (subgoal 4).

#### Continuous Monitoring and Model Updating

It is likely that our models, particularly those based on a larger number of input variables, do not reach their full performance plateau when trained on the described datasets. Consequently, retraining the model using data collected after system implementation is expected to further improve predictive performance. However, this process requires careful oversight, as the inclusion of erroneous or low-quality data could degrade rather than enhance model performance. To mitigate this risk, only observations and estimations that have a corresponding directly measured VO_2max_ value within plus or minus 7 days of the estimated value will be used for retraining.

To monitor and control data drift, we will retain 20% of all incoming data in a holdout monitoring set, in addition to the original test set from this study. This subset will be stratified by center’s location to ensure geographic and operational diversity. This design allows us to detect both temporal drift and center-specific drift. Drift will be quantified using multiple performance metrics, including root mean squared error, mean absolute percentage error, SEE, and *R*-squared score.

Furthermore, we will cluster incoming observations and identify potential out-of-distribution samples. These cases will be flagged and manually reviewed before inclusion in either the training or evaluation datasets. Model performance will also be continuously assessed across clinically and demographically relevant subgroups, including sex, age groups, and BMI categories, to detect potential bias or subgroup-specific degradation in performance.

All monitoring procedures, performance metrics, and visualizations will be integrated into a dedicated dashboard accessible to system administrators.

Model retraining may present regulatory challenges in relation to the European Union Medical Device Regulation (MDR) and CE marking, as substantive changes to the model may require recertification. For this reason, retraining will initially be limited to scenarios where the application is marketed as a health or wellness application rather than a regulated clinical decision-support tool. The initial release will therefore be positioned as a health application, with the longer-term objective of potential reclassification as a clinical tool, subject to regulatory approval.

### Ethical Considerations

First, this project relies on data from various research studies and a clinical registry, both of which have been approved for this purpose by the Regional Committees for Medical and Health Research Ethics South East, Norway (REK ID: 2013/1849, 2016/1414, 2017/88, and 2009/1699 [38]) or the Norwegian Data Protection Authority (ID: 17/00433‐2/SBO [39]). Further, 2 of the studies are published in international research journals [19,26].

Second, all data from the included studies and registers are either pseudonymized or anonymized, and the welfare and integrity of participants will be safeguarded through the careful selection of variables that minimize the risk of reidentification. The variables do not include easily recognizable external background information, such as place of residence or occupation, which could increase the likelihood of retrospective identification. However, variables such as height, weight, and VO_2max_ may, in rare cases, allow for reidentification at extreme values. To address this, it may be necessary to exclude extreme values (outliers) within these categories.

Third, written informed consent was obtained from all patients, with the intention of using the data for research.

Fourth, a “motivated intruder” test will be conducted to assess the potential for data reidentification. While it is impossible to eliminate the risk of reidentification with absolute certainty, these measures are designed to significantly mitigate such risks. Furthermore, the project uses systems that have undergone rigorous risk assessment and are approved for handling sensitive data, ensuring adherence to the highest standards of data security and privacy.

Fifth, studies 1, 2, and 4 are fully anonymized, with the linking keys to patients’ identities permanently deleted. As a result, the Norwegian Agency for Shared Services in Education and Research granted approval for the use of these data in the project. Studies 3 and 5 are pseudonymized; however, the linking keys to patients’ identities have not been deleted. Consequently, the project applied to the Norwegian Directorate of Health for an exemption from the requirement to obtain patient consent for the use of the data. For Study 4, broad consent has been obtained from the patients, allowing the inclusion of these data in the project.

Sixth, to avoid recognition in the data material from the southeast region of Norway (VHT and NIH), this study included patients from the central region of Norway, Møre and Romsdal (MRI).

Seventh, to ensure rigorous adherence to ethical standards, the project applied to the Regional Committees for Medical and Health Research Ethics South East, Norway (REK ID 741918). They determined that the project falls outside the scope of the Health Research Act, cf Section II. However, clinical trials involving medical devices may fall under the purview of the European Union MDR 2017/745. The MDR explicitly states that applications, software, and devices incorporating AI can be classified as medical devices. Consequently, it may be necessary to apply following the project’s completion to facilitate the implementation of the web application as a certified medical device at the end of this project.

This project is registered at ClinicalTrials.gov (identifier: NCT07011108). VHT is the coordinating center. Responsibility for data management lies with each center that is the data controller for its own data set. VHT will have access to the final data set. No financial compensation, incentives, or other forms of remuneration will be provided to participants.

## Results

The project was awarded salary funding in two rounds from Vestfold Hospital (October and December 2023) and additional salary funding from the Research Council of Norway (October 2024). In addition, the project received allocated supervision hours from InnoMed Norway (April 2024). As of submission (February 2026), the dataset includes 1279 adults with severe obesity and 2623 VO₂max tests collected before, during, and after lifestyle interventions conducted between 2013 and 2025.

For the retrospective component of the study, ethical approval has been obtained to use 2 of the 5 databases for initial model development. Ethics review for the remaining 3 databases is currently pending. Upon completion of the initial model development phase, ethical approval will be sought to conduct the prospective investigation.

Data analysis is ongoing. A web-based application has been developed, and the first and second rounds of formative user testing have been conducted with adult patients with overweight and obesity and health care personnel. The first scientific publication of the clinically relevant machine learning model is expected in 2026, and the overall project is expected to be completed in 2028.

## Discussion

### Principal Findings

Patients with obesity have low relative VO_2max_ compared to normal-weight people. Therefore, patients with obesity are outside the range of validity of the statistical models trained on normal-weight people [13,19,20], which results in an imprecise estimate of CRF for patients with obesity using statistical models trained on normal-weight people. To address this problem, a retrospective study will be conducted, using anonymized datasets from 4 research studies and 1 clinical quality registry.

Furthermore, to address the—often seen—performance drop by ML models on data gathered in a different context than training data, this study plans to validate the model from the previous step (retrospective part) prospectively.

### Strengths and Limitations

The ML-based estimation will be implemented as a clinical tool in the form of a web application, designed to assist health care professionals across various settings, including hospitals, rehabilitation centers, primary care services, and general practice. This tool aims to facilitate faster, more precise, and personalized treatment while alleviating the burden on health care services by obviating the need for direct physical measurements of VO_2max_. Furthermore, the integration of advanced technologies and optimized workflows has the potential to enhance efficiency, allowing health care professionals to allocate more time to patient-centered tasks or mitigate waiting times. Additionally, the web application seeks to empower both patients and the general population by fostering greater autonomy in managing their health, enhancing health literacy, and promoting engagement in preventive health measures.

### Stakeholder Involvement

Further, the primary health care services play a pivotal role in strengthening health competencies at the municipal level and alleviating the demand for specialized health care services. Therefore, to ensure the successful implementation of this innovation and research initiative, the primary health care sector is actively engaged in its development, contributing to its relevance, feasibility, and integration into existing health care frameworks. Furthermore, the active involvement of patients throughout the entire study is considered to be essential for its successful development and implementation.

### Conclusion

To conclude, the project members anticipate that using longitudinal, real-world clinical data from a uniquely characterized population with obesity will enable the development and validation of a clinically relevant ML model for estimating CRF that provides a more practical alternative to current assessment methods, thereby reducing time, costs, and procedural burden in clinical practice. This may enhance accessibility and reinforce CRF as a valuable diagnostic and prognostic assessment of cardiovascular health for individuals with obesity. Such a predictive tool may also facilitate more frequent CRF assessments, enabling more detailed monitoring of changes in CRF over time. The broader aim of this project is to achieve national and international significance, contributing to improved patient outcomes and supporting health care professionals globally.

To our knowledge, VHT, MRI, and NIH are likely the only institutions in Norway that systematically assess direct physical measurements of VO_2max_ as part of both research projects and clinical treatments for obese patients. This gives us a unique database for the development of ML models for CRF estimation for this specific patient population. To our knowledge, this study will be the first attempt to estimate CRF in patients with obesity, based on clinical parameters, using ML. The project members expect that the project will hold significant societal value by contributing to improved public health and advancing knowledge related to the management of severe obesity. Obesity represents a major public health challenge with substantial individual, societal, and economic consequences. By investigating physiological responses and cardiorespiratory fitness outcomes in patients with severe obesity, this study may provide evidence that informs clinical practice, supports individualized treatment strategies, and contributes to more effective health care resource allocation.

### Communication

The project will be disseminated through professional hospital channels and to relevant stakeholders nationally and internationally. The Norwegian Association for People with Obesity will support accessible communication to the patient group through its established channels. Results will be disseminated through national and international media, presented at major scientific conferences, and published in peer-reviewed journals.

## Supplementary material

10.2196/85069 Checklist 1 SPIRIT (Standard Protocol Items: Recommendations for Interventional Trials)-artificial intelligence checklist.

## Abbreviation

AI: artificial intelligence
CONSORT: Consolidated Standards of Reporting Trials
CRF: cardiorespiratory fitness
MDR: Medical Device Regulation
ML: machine learning
MRI: Muritunet Rehabilitation Institution
NIH: Norwegian School of Sport Sciences
SEE: standard error of estimate
SPIRIT: Standard Protocol Items: Recommendations for Interventional Trials
VHT: Vestfold Hospital Trust
VO_2max_: maximum volume of oxygen (mL/min)

## References

[1] (2021). Obesity. World Health Organization.

[2] (2019). Overvekt og fedme i norge: omfang, utvikling og samfunnskostnader [Report in Norwegian]. https://menon.no/uploads/images/2019-09-Overvekt-og-fedme-i-Norge.pdf.

[3] Laukkanen JA, Lakka TA, Rauramaa R (2001). Cardiovascular fitness as a predictor of mortality in men. Arch Intern Med.

[4] Kodama S, Saito K, Tanaka S (2009). Cardiorespiratory fitness as a quantitative predictor of all-cause mortality and cardiovascular events in healthy men and women: a meta-analysis. JAMA.

[5] Wing RR, Jakicic J, Neiberg R (2007). Fitness, fatness, and cardiovascular risk factors in type 2 diabetes: look ahead study. Med Sci Sports Exerc.

[6] Lee D, Artero EG, Sui X, Blair SN (2010). Mortality trends in the general population: the importance of cardiorespiratory fitness. J Psychopharmacol.

[7] Kokkinos P, Faselis C, Franklin B (2019). Cardiorespiratory fitness, body mass index and heart failure incidence. Eur J Heart Fail.

[8] Kent M (2006). The Oxford Dictionary of Sports Science & Medicine.

[9] Wammer F, Haberberger A, Linge AD, Myklebust TÅ, Vemøy S, Hoff DAL (2022). Lifestyle modification for weight loss: effects on cardiorespiratory capacity in patients with class II and class III obesity. Obes Sci Pract.

[10] Gaesser GA, Angadi SS (2021). Obesity treatment: weight loss versus increasing fitness and physical activity for reducing health risks. iScience.

[11] Weeldreyer NR, De Guzman JC, Paterson C, Allen JD, Gaesser GA, Angadi SS (2025). Cardiorespiratory fitness, body mass index and mortality: a systematic review and meta-analysis. Br J Sports Med.

[12] McArdle WD, Katch FI, Katch VL (2010). Exercise Physiology: Nutrition, Energy, and Human Performance.

[13] Ashfaq A, Cronin N, Müller P (2022). Recent advances in machine learning for maximal oxygen uptake (VO2 max) prediction: a review. Inform Med Unlocked.

[14] Petelczyc M, Kotlewski M, Bruhn S, Weippert M (2023). Maximal oxygen uptake prediction from submaximal bicycle ergometry using a differential model. Sci Rep.

[15] Cink RE, Thomas TR (1981). Validity of the Astrand-Ryhming nomogram for predicting maximal oxygen intake. Br J Sports Med.

[16] Helgerud J, Haglo H, Hoff J (2022). Prediction of VO2max from submaximal exercise using the smartphone application Myworkout GO: validation study of a digital health method. JMIR Cardio.

[17] Nes BM, Janszky I, Vatten LJ, Nilsen TIL, Aspenes ST, Wisløff U (2011). Estimating V·O 2peak from a nonexercise prediction model: the HUNT study, Norway. Med Sci Sports Exerc.

[18] Alzamer H, Abuhmed T, Hamad K (2021). A short review on the machine learning-guided oxygen uptake prediction for sport science applications. Electronics.

[19] Berge J, Støren Ø, Hertel JK, Gjevestad E, Småstuen MC, Hjelmesæth J (2019). Associations between cardiorespiratory fitness and weight loss in patients with severe obesity undergoing an intensive lifestyle intervention program: retrospective cohort study. BMC Endocr Disord.

[20] Nevill AM, Cooke CB (2017). The dangers of estimating V˙O2max using linear, nonexercise prediction models. Med Sci Sports Exerc.

[21] (2024). Rules for trustworthy artificial intelligence in the EU. EUR‑Lex – Access to European Union law.

[22] Liu X, Rivera SC, Moher D, Calvert MJ, Denniston AK, SPIRIT-AI and CONSORT-AI Working Group (2020). Reporting guidelines for clinical trial reports for interventions involving artificial intelligence: the CONSORT-AI extension. Lancet Digit Health.

[23] Collins GS, Moons KGM, Dhiman P (2024). TRIPOD+AI statement: updated guidance for reporting clinical prediction models that use regression or machine learning methods. BMJ.

[24] Eysenbach G, CONSORT-EHEALTH Group (2011). CONSORT-EHEALTH: improving and standardizing evaluation reports of web-based and mobile health interventions. J Med Internet Res.

[25] Rivera SC, Liu X, Chan AW, Denniston AK, Calvert MJ, SPIRIT-AI and CONSORT-AI Working Group (2020). Guidelines for clinical trial protocols for interventions involving artificial intelligence: the SPIRIT-AI extension. BMJ.

[26] Berge J, Hjelmesaeth J, Hertel JK (2021). Effect of aerobic exercise intensity on energy expenditure and weight loss in severe obesity: a randomized controlled trial. Obesity (Silver Spring).

[27] Ware JE, Sherbourne CD (1992). The MOS 36-item short-form health survey (SF-36). I. Conceptual framework and item selection. Med Care.

[28] Ware JE (2000). SF-36 health survey update. Spine (Phila Pa 1976).

[29] Kolotkin RL, Crosby RD, Kosloski KD, Williams GR (2001). Development of a brief measure to assess quality of life in obesity. Obes Res.

[30] Patrick DL, Bushnell DM, Rothman M (2004). Performance of two self-report measures for evaluating obesity and weight loss. Obes Res.

[31] Niero M, Martin M, Finger T (2002). A new approach to multicultural item generation in the development of two obesity-specific measures: the obesity and weight loss quality of life (OWLQOL) questionnaire and the weight-related symptom measure (WRSM). Clin Ther.

[32] Loge JH, Kaasa S, Hjermstad MJ, Kvien TK (1998). Translation and performance of the Norwegian SF-36 Health Survey in patients with rheumatoid arthritis. I. Data quality, scaling assumptions, reliability, and construct validity. J Clin Epidemiol.

[33] Huppertz-Hauss G, Lie Høivik M, Jelsness-Jørgensen LP (2016). Health-related quality of life in patients with inflammatory bowel disease 20 years after diagnosis: results from the IBSEN study. Inflamm Bowel Dis.

[34] Garratt AM, Stavem K (2017). Measurement properties and normative data for the Norwegian SF-36: results from a general population survey. Health Qual Life Outcomes.

[35] Loge JH, Kaasa S (1998). Short form 36 (SF-36) health survey: normative data from the general Norwegian population. Scand J Soc Med.

[36] Karlsen TI, Tveitå EK, Natvig GK, Tonstad S, Hjelmesæth J (2011). Validity of the SF-36 in patients with morbid obesity. Obes Facts.

[37] Riley RD, Snell KI, Ensor J (2019). Minimum sample size for developing a multivariable prediction model: PART II - binary and time-to-event outcomes. Stat Med.

[38] REK.

[39] Datatilsynet.

