# Supplementary material for: Machine Learning for Estimating Cardiorespiratory Fitness in Patients With Obesity: Protocol for a Retrospective and Prospective Multicenter Cohort Study
Source: JMIR Res Protoc. 2026 Mar 2;15:e85069. doi: 10.2196/85069 (PMC12954685; doi:10.2196/85069)
Supplement: Checklist 1 [file resprot-v15-e85069-s001.pdf]

SPIRIT-AI Checklist: Recommended items to address in a protocol and related documents for clinical trials evaluating AI interventions

Section		SPIRIT 2013 Item[a]	SPIRIT-AI Item		Addressed on Page No [b]
Administrative Information					
Title	1	Descriptive title identifying the study design, population, interventions, and, if applicable, trial acronym	SPIRIT-AI 1(i) Elaboration	Indicate that the intervention involves artificial intelligence / machine learning and specify the type of model.	1
			SPIRIT-AI 1(ii) Elaboration	Specify the intended use of the AI intervention.	1
Trial registration	2a	Trial identifier and registry name. If not yet registered, name of intended registry			2
	2b	All items from the World Health Organization Trial Registration Data Set			Throughout protocol
Protocol version	3	Date and version identifier			2
Funding	4	Sources and types of financial, material, and other support			20
Roles and responsibilities	5a	Names, affiliations, and roles of protocol contributors			1
	5b	Name and contact information for the trial sponsor			N/A
	5c	Role of study sponsor and funders, if any, in study design; collection, management, analysis, and interpretation of data; writing of the report; and the decision to submit the report for publication, including whether they will have ultimate authority over any of these activities			N/A
	5d	Composition, roles, and responsibilities of the coordinating centre, steering committee, endpoint adjudication committee, data management team, and other individuals or groups overseeing the trial, if applicable (see Item 21a for data monitoring committee)			20
Introduction					
Background and rationale	6a	Description of research question and justification for undertaking the trial, including summary of relevant studies (published and unpublished) examining benefits and harms for each intervention	SPIRIT-AI 6a (i) Extension	Explain the intended use of the AI intervention in the context of the clinical pathway, including its purpose and its intended users (e.g. healthcare professionals, patients, public).	3-4
			SPIRIT-AI 6a (ii) Extension	Describe any pre-existing evidence for the AI intervention.	3-4

	6b	Explanation for choice of comparators			3-4
Objectives	7	Specific objectives or hypotheses			4
Trial design	8	Description of trial design including type of trial (eg, parallel group, crossover, factorial, single group), allocation ratio, and framework (eg, superiority, equivalence, noninferiority, exploratory)			5
Methods: Participants, Interventions and Outcomes					
Study setting	9	Description of study settings (eg, community clinic, academic hospital) and list of countries where data will be collected. Reference to where list of study sites can be obtained	SPIRIT-AI 9 Extension	Describe the onsite and offsite requirements needed to integrate the AI intervention into the trial setting.	13
Eligibility criteria	10	Inclusion and exclusion criteria for participants. If applicable, eligibility criteria for study centres and individuals who will perform the interventions (eg, surgeons, psychotherapists)	SPIRIT-AI 10 (i) Elaboration	State the inclusion and exclusion criteria at the level of participants.	5
			SPIRIT-AI 10 (ii) Extension	State the inclusion and exclusion criteria at the level of the input data.	6
Interventions	11a	Interventions for each group with sufficient detail to allow replication, including how and when they will be administered	SPIRIT-AI 11a (i) Extension	State which version of the AI algorithm will be used.	N/A
			SPIRIT-AI 11a (ii) Extension	Specify the procedure for acquiring and selecting the input data for the AI intervention.	6-7
			SPIRIT-AI 11a (iii) Extension	Specify the procedure for assessing and handling poor quality or unavailable input data.	5
			SPIRIT-AI 11a (iv) Extension	Specify whether there is human-AI interaction in the handling of the input data, and what level of expertise is required for users.	12
			SPIRIT-AI 11a (v) Extension	Specify the output of the AI intervention.	12
			SPIRIT-AI 11a (vi) Extension	Explain the procedure for how the AI intervention's output will contribute to decision-making or other elements of clinical practice.	12
	11b	Criteria for discontinuing or modifying allocated interventions for a given trial participant (eg, drug dose change in response to harms, participant request, or improving/worsening disease)			
	11c	Strategies to improve adherence to intervention protocols, and any procedures for monitoring adherence (eg, drug tablet return, laboratory tests)			
	11d	Relevant concomitant care and interventions that are permitted or prohibited during the trial			
Outcomes	12	Primary, secondary, and other outcomes, including the specific measurement variable (eg, systolic blood pressure), analysis metric (eg, change from baseline, final value, time to event), method of aggregation (eg, median, proportion), and time point for each outcome. Explanation of the clinical relevance of chosen efficacy and harm outcomes is strongly recommended			12

Participant timeline	13	Time schedule of enrolment, interventions (including any run-ins and washouts), assessments, and visits for participants. A schematic diagram is highly recommended (see Figure)			N/A
Sample size	14	Estimated number of participants needed to achieve study objectives and how it was determined, including clinical and statistical assumptions supporting any sample size calculations			13-14
Recruitment	15	Strategies for achieving adequate participant enrolment to reach target sample size			N/A
Methods: Assignment of Interventions (For Controlled Trials)					
Sequence generation	16A	Method of generating the allocation sequence (eg, computer-generated random numbers), and list of any factors for stratification. To reduce predictability of a random sequence, details of any planned restriction (eg, blocking) should be provided in a separate document that is unavailable to those who enrol participants or assign interventions			
Allocation concealment mechanism	16b	Mechanism of implementing the allocation sequence (eg, central telephone; sequentially numbered, opaque, sealed envelopes), describing any steps to conceal the sequence until interventions are assigned			
Implementation	16c	Who will generate the allocation sequence, who will enrol participants, and who will assign participants to interventions			
Blinding (masking)	17a	Who will be blinded after assignment to interventions (eg, trial participants, care providers, outcome assessors, data analysts), and how			
	17b	If blinded, circumstances under which unblinding is permissible, and procedure for revealing a participant’s allocated intervention during the trial			
Methods: Data Collection, Management, And Analysis					
Data collection methods	18a	Plans for assessment and collection of outcome, baseline, and other trial data, including any related processes to promote data quality (eg, duplicate measurements, training of assessors) and a description of study instruments (eg, questionnaires, laboratory tests) along with their reliability and validity, if known. Reference to where data collection forms can be found, if not in the protocol			6-7
	18b	Plans to promote participant retention and complete follow-up, including list of any outcome data to be collected for participants who discontinue or deviate from intervention protocols			N/A
Data management	19	Plans for data entry, coding, security, and storage, including any related processes to promote data quality (eg, double data entry; range checks for data values). Reference to where details of data management procedures can be found, if not in the protocol			15-17

Statistical methods	20a	Statistical methods for analysing primary and secondary outcomes. Reference to where other details of the statistical analysis plan can be found, if not in the protocol			12-17
	20b	Methods for any additional analyses (eg, subgroup and adjusted analyses)			N/A
	20c	Definition of analysis population relating to protocol non-adherence (eg, as randomised analysis), and any statistical methods to handle missing data (eg, multiple imputation)			15
Methods: Monitoring					
Data monitoring	21a	Composition of data monitoring committee (DMC); summary of its role and reporting structure; statement of whether it is independent from the sponsor and competing interests; and reference to where further details about its charter can be found, if not in the protocol. Alternatively, an explanation of why a DMC is not needed			
	21b	Description of any interim analyses and stopping guidelines, including who will have access to these interim results and make the final decision to terminate the trial			
Harms	22	Plans for collecting, assessing, reporting, and managing solicited and spontaneously reported adverse events and other unintended effects of trial interventions or trial conduct	SPIRIT-AI 22 Extension	Specify any plans to identify and analyse performance errors. If there are no plans for this, explain why not.	
Auditing	23	Frequency and procedures for auditing trial conduct, if any, and whether the process will be independent from investigators and the sponsor			
Ethics and Dissemination					
Research ethics approval	24	Plans for seeking research ethics committee/institutional review board (REC/IRB) approval			17-18
Protocol amendments	25	Plans for communicating important protocol modifications (eg, changes to eligibility criteria, outcomes, analyses) to relevant parties (eg, investigators, REC/IRBs, trial participants, trial registries, journals, regulators)			16
Consent or ascent	26a	Who will obtain informed consent or assent from potential trial participants or authorised surrogates, and how (see Item 32)			17
	26b	Additional consent provisions for collection and use of participant data and biological specimens in ancillary studies, if applicable			Not applicable
Confidentiality	27	How personal information about potential and enrolled participants will be collected, shared, and maintained in order to protect confidentiality before, during, and after the trial			17
Declaration of interests	28	Financial and other competing interests for principal investigators for the overall trial and each study site			20-21

Access to data	29	Statement of who will have access to the final trial dataset, and disclosure of contractual agreements that limit such access for investigators	SPIRIT-AI 29 Extension	State whether and how the AI intervention and/or its code can be accessed, including any restrictions to access or re-use.	20
Ancillary and post-trial care	30	Provisions, if any, for ancillary and post-trial care, and for compensation to those who suffer harm from trial participation			Not applicable
Dissemination policy	31a	Plans for investigators and sponsor to communicate trial results to participants, healthcare professionals, the public, and other relevant groups (eg, via publication, reporting in results databases, or other data sharing arrangements), including any publication restrictions			19
	31b	Authorship eligibility guidelines and any intended use of professional writers			21
	31c	Plans, if any, for granting public access to the full protocol, participant-level dataset, and statistical code			Not applicable
Appendices					
Informed consent materials	32	Model consent form and other related documentation given to participants and authorised surrogates			N/A
Biological specimens	33	Plans for collection, laboratory evaluation, and storage of biological specimens for genetic or molecular analysis in the current trial and for future use in ancillary studies, if applicable			Not applicable

[a] It is strongly recommended that this checklist be read in conjunction with the SPIRIT 2013 Explanation & Elaboration for important clarification on the items.

[b] Indicates page numbers to be completed by authors during protocol development.
